# Circuit Investigations With Open-Source Miniaturized Microscopes: Past, Present and Future

**DOI:** 10.3389/fncel.2019.00141

**Published:** 2019-04-05

**Authors:** Daniel Aharoni, Tycho M. Hoogland

**Affiliations:** ^1^Department of Neurology, David Geffen School of Medicine, University of California, Los Angeles, Los Angeles, CA, United States; ^2^Department of Neuroscience, Erasmus Medical Center, Rotterdam, Netherlands; ^3^Netherlands Institute for Neuroscience, Royal Netherlands Academy of Arts and Sciences, Amsterdam, Netherlands

**Keywords:** miniscope, behavior, freely moving animals, open-source, miniaturization, 3D printing, systems neurobiology

## Abstract

The ability to simultaneously image the spatiotemporal activity signatures from many neurons during unrestrained vertebrate behaviors has become possible through the development of miniaturized fluorescence microscopes, or miniscopes, sufficiently light to be carried by small animals such as bats, birds and rodents. Miniscopes have permitted the study of circuits underlying song vocalization, action sequencing, head-direction tuning, spatial memory encoding and sleep to name a few. The foundation for these microscopes has been laid over the last two decades through academic research with some of this work resulting in commercialization. More recently, open-source initiatives have led to an even broader adoption of miniscopes in the neuroscience community. Open-source designs allow for rapid modification and extension of their function, which has resulted in a new generation of miniscopes that now permit wire-free or wireless recording, concurrent electrophysiology and imaging, two-color fluorescence detection, simultaneous optical actuation and read-out as well as wide-field and volumetric light-field imaging. These novel miniscopes will further expand the toolset of those seeking affordable methods to probe neural circuit function during naturalistic behaviors. Here, we will discuss the early development, present use and future potential of miniscopes.

## Introduction

The past 5 years have seen a flurry of open-source development with respect to miniaturized fluorescence microscopes (miniscopes) for neuroscience applications, further advancing already existing technology and extending access to a much broader scientific user base (Cai et al., 2016; Liberti et al., 2016, 2017; Jacob et al., 2018b; Juneau et al., 2018; Liang et al., 2018; Scott et al., 2018; Skocek et al., 2018; Zhang et al., 2018). The open-source nature of ongoing miniscope projects allows for the rapid sharing of designs, modifications and ideas within the neuroscience community. This has led to a dramatic acceleration of innovation resulting in miniscopes capable of controlling the activity of cell populations while imaging neural activity in freely moving vertebrates, distinguishing distinct cell populations using two-color capable versions (Jacob et al., 2018b; Leman et al., 2018), mapping out larger fields-of-view (Leman et al., 2018), imaging volumes at video frame rates (Skocek et al., 2018) and simultaneously recording animal vocalization (Liberti et al., 2017) or head acceleration (De Groot et al., 2018). In this review article, we summarize and compare the different ongoing open-source developments and their potential for neuroscientific inquiries. The main driving force behind the development of miniaturized microscopes is first and foremost their ability to record activity from many neurons with a defined topology in an animal that is unrestrained and can display its natural innate behavior. Factors affecting behavior such as elevated stress levels in head-fixed animals and the lack of vestibular input are likely to affect recorded neural activity patterns and thus the insights that can be inferred about brain function in more natural states (Thurley and Ayaz, 2017). In addition, miniscopes provide a window into neural activity underlying behavior across the large array of behavioral assays developed over the past decades (Morris, 1984; Graeff et al., 1998; Nadler et al., 2004; Gomez-Marin et al., 2014). As a result, accurate behavioral tracking and quantification (Matsumoto et al., 2013; Wiltschko et al., 2015; Mathis et al., 2018; Mimica et al., 2018; Pereira et al., 2019) become essential in understanding how activity recorded from targeted regions can be correlated with behavior. Furthermore, the ability to record from more than one region either by sampling from larger imaging areas or by using multiple imaging devices with a reduced footprint and weight (De Groot et al., 2018) should uncover how inter-regional signaling plays out during more natural behaviors.

## Miniaturized One-Photon Excitation Microscopes

**Figure 1:**
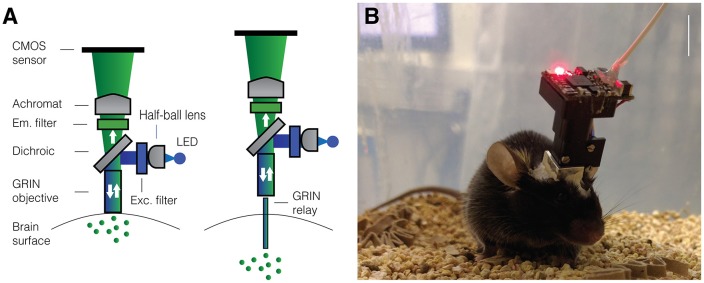
Miniaturized one-photon excitation microscope design. **(A)** A typical design for a miniaturized one-photon excitation microscope used in combination with gradient refractive index (GRIN) lenses. It is comprised of an excitation LED light source, half-ball lens light collimator, excitation filter (Exc. filter) and dichroic mirror for reflecting excitation light down toward the specimen and transmitting emitted fluorescence up to the imaging detector. Emitted light is focused onto a CMOS imaging sensor using an achromatic lens after passing through an emission filter (Em. filter). The use of GRIN lenses permits imaging from both superficial (left) or deep-lying (right) brain structures. Adjustment of the focal plane in the specimen is achieved by moving the image sensor towards or away from the achromatic lens. For superficial imaging, the objective GRIN lens is placed directly on the brain surface, for deep brain imaging the objective GRIN lens is mounted inside the scope and combined with a thinner relay GRIN lens that is implanted into the brain to image from cells transduced with a fluorescent activity reporter (green dots). **(B)** An open-source first-generation UCLA Miniscope, which is mounted, *via* a baseplate, on a mouse for the duration of the recording session. Mice carry these 3-g miniscopes without any overt effects on overall behavior, although cabled versions may affect social interactions with other mice. Scale bar ~10 mm.

A wide variety of approaches have been tried and tested to image neural activity in freely behaving animals. Many of the initial systems developed were optical fiber-based (Helmchen et al., 2001; Helmchen, 2002; Göbel et al., 2004; Flusberg et al., 2005, 2008)—with excitation light coupled in and fluorescence light collected away from the microscope housing—preceding the development of a head-mounted miniaturized microscope where fluorescence excitation and detection were combined onboard the microscope housing (Ghosh et al., 2011). The components of such a miniature microscope overcame cost limitations of table-top lasers and expensive detectors and instead made use of relatively accessible technologies such as CMOS imaging sensors, LEDs—as light source—, off-the-shelf optical components and Gradient Refractive Index (or GRIN) lenses. A typical design for such a miniaturized microscope is shown in Figure 1. The optical path is very similar to that of a conventional, wide-field fluorescence microscope with the notable difference being the use of a single or set of GRIN lenses. GRIN lenses provide an optical interface to the brain benefiting from their short working distance, flat bottom and range of lengths and diameters. For superficial brain imaging, a single objective GRIN lens is placed directly on the brain surface. For access to deeper areas, an objective GRIN lens is combined with a smaller diameter relay GRIN lens, implanted above the neurons of interest, typically at the expense of a more limited field-of-view. In comparison to two-photon excitation, one-photon excitation is particularly susceptible to out-of-focus fluorescence and poor optical sectioning. Despite this apparent drawback, it has proven possible to extract signals from individual neurons using analytical techniques, including a combination of principal and independent component analysis (PCA/ICA) and more recently constrained non-negative matrix factorization (CNMF) with an added term to model a time-varying fluorescence background signal (Mukamel et al., 2009; Lu et al., 2018; Zhou et al., 2018). Thus, miniaturized head-mounted fluorescence microscopes leverage the use of cheap components while still enabling cellular-resolution imaging in awake behaving animals. The availability of such miniscopes through commercial vendors has already led to a multitude of studies that have provided better insights into neural circuit activity underlying action sequencing, anxiety, vocalization—in birds—, social memories and sleep (Markowitz et al., 2015; Okuyama et al., 2016; Klaus et al., 2017; Chen et al., 2018; Jimenez et al., 2018). The release of open-source building plans for miniscopes has led to an even broader application of this technology in the past few years and will likely provide a consolidated platform for future iterations of tools to understand how topologically defined activity patterns in the brain contribute to behavior (Aharoni et al., 2019). The increased application of miniscopes to image neural activity in awake behaving animals would not have been possible without advances in the development of fluorescent activity reporters. For example, derivatives of the genetically encoded calcium indicator (GECI) protein GCaMP (Chen et al., 2013) have permitted cell-specific targeting of activity sensors in combination with improved detection of neural activity and subcellular targeting of such sensors (Dana et al., 2016). Moreover, strides are being made to develop more sensitive voltage sensors that can be targeted with cellular specificity *in vivo* (Bando et al., 2019; Quicke et al., 2019). Fluorescent sensors for glutamate and dopamine transients have also become available enabling imaging neurotransmitter release in awake behaving animals (Marvin et al., 2018; Patriarchi et al., 2018). Thus, various aspects of circuit level function can now be investigated using miniscopes during unrestrained behavior. There are caveats associated with using miniaturized fluorescence microscopes. The use of GRIN lenses to access deeper-lying structures requires insertion of lenses into the brain, which is associated with tissue damage, despite some indications that careful surgical procedures may limit effects on behavior—at least in mice (Lee et al., 2016). In any case, care should be taken to ensure that inflammatory responses have abated after a craniotomy is made for access to the brain and GRIN lenses have been inserted (Bocarsly et al., 2015). Moreover, out of cost considerations as well as ease of use, most miniaturized fluorescence microscopes use single photon excitation, which limits optical sectioning and makes it harder to extract signals from a single source. The latter necessitates *post hoc* algorithmic extraction of signals that incorporate models of out-of-focus fluorescence, which themselves are based on assumptions. Despite these clear shortcomings, miniscopes and their open-source versions have opened up the study of large ensembles of neurons during naturalistic behavior. Since cost is no longer a limitation to perform such experiments, it is a good time to provide an overview of the accessibility and applicability of the open-source projects that have made this technology accessible. The requirement for behavioral mapping, direct control over circuit level activity as well as more fine-grained read-out of sub-populations of neurons within a circuit has led several labs to develop new iterations of miniscopes to meet these needs. We will therefore not only discuss currently existing open-source designs but elaborate on future iterations and technologies that are aimed at improving and expanding overall functionality of miniscopes.

## Open-Source Miniscope Projects

### UCLA Miniscope

**Figure 2:**
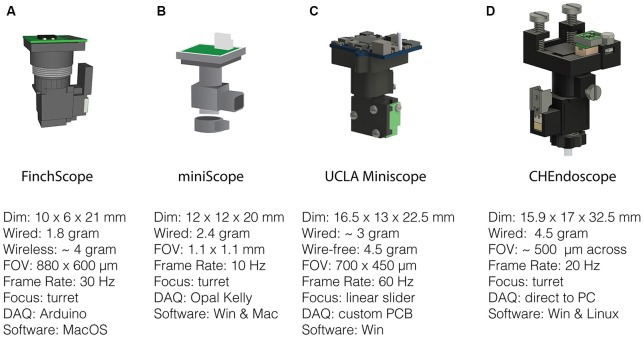
Open-source miniscopes released in the public domain. **(A)** FinchScope (https://github.com/gardner-lab/FinchScope), image credit: W.A. Liberti III. **(B)** miniScope (https://github.com/giovannibarbera/miniscope_v1.0). **(C)** UCLA Miniscope (http://www.miniscope.org). **(D)** CHEndoscope (https://github.com/jf-lab/chendoscope), image credit: A. Jacob, Josselyn lab.

With several labs having pursued unique versions of miniaturized microscopes (Figure 2) that were subsequently released into the public domain, the UCLA Miniscope project has probably been the most impactful in terms of its reach, with over 400 labs around the world building and applying these imaging devices in their research over the past 3 years. This broad dissemination was achieved through online documentation and tutorials on part procurement, assembly and experimental application as well as through numerous in person workshops (for details, please visit: http://miniscope.org). The first generation of UCLA Miniscopes, which were first used to show that memories formed close in time show a greater overlap of CA1 neural ensembles (Cai et al., 2016), can be built at a fraction of the cost of their commercial counterparts, weighs about 3 g, has a field-of-view of 700 by 450 μm (752 by 480 pixels, 6 μm pixel size), permits single channel fluorescence imaging up to a maximum rate of 60 Hz and concurrent monitoring of behavior with an additional USB camera. The optical design includes an achromat lens (Edmund Optics 49-923) which focuses nearly collimated light from an objective GRIN lens onto a CMOS sensor and a set of custom-diced optical filters (Chroma ET470/40x, T495lpxr, ET525/50 m). The housing is CNC machined out of black Delrin and custom electronics are used to control and read-out the excitation LED light source (Luxeon LXML-PB01-0030) and CMOS imaging sensor (On Semiconductor MT9V032C12STM, dynamic range > 55 dB). This CMOS imaging sensor was chosen due to its relatively small size at the time of design, low cost, large pixel size and availability at low order quantities. In addition, a similar CMOS imaging sensor had been previously shown to be able to resolve the fluorescent dynamics of GECIs (Ghosh et al., 2011). For superficial imaging, the objective GRIN lens is implanted on top of the brain surface. A metal base plate attached to the skull with dental cement allows mounting of the miniscope. For imaging deeper brain regions, a second thinner relay GRIN lens may be implanted. In this case the objective GRIN lens is mounted inside the miniscope and positioned above the relay lens to bring cells into focus (Figure 1). Focusing occurs through a linear slider that is manually adjusted in height, preserving the orientation of neurons between imaging sessions. Unique to the UCLA Miniscope project is the cabling connecting the head-mounted scope and the off-board DAQ hardware. Power, communication, and data transmission are achieved through a single, flexible coaxial cable in conjunction with supporting hardware (TI DS90UB913A/DS90UB914A and Power-Over-Coax filters). With a total cable diameter down to 0.3 mm (Molex 100065-0023) and compatibility with passive, low torque commutators, this design minimizes the impact of cabling on animal behavior.

A wire-free version of the UCLA Miniscope (4.5 g) has also been developed and used to record CA1 place cells during maze navigation in epileptic mice (Shuman et al., 2018). This system incorporates a lithium-polymer battery (~1 g) for power, a MicroSD card for local data storage (~0.5 g) and a power efficient imaging sensor (E2V JADE EV76C454). In order to keep power consumption low, data is recorded at a resolution of 320 by 320 pixels using two times pixel sub-sampling. Although heavier than its wired counterpart, the use of a wire-free design resulted in social behavior comparable to that of mice without a Miniscope, whereas the lighter wired version in fact, showed reduced social exploration (Supplemental Data in Shuman et al., 2018).

### FinchScope

The FinchScope project initiated at Boston University (Liberti et al., 2017) resulted in an open-source microscope around the same time as the UCLA Miniscope. This project uses high-resolution 3D printed parts (low-fluorescent resins FGPBLK01 and FGPBLK02 on a benchtop Form 2 printer) and cheap, off-the-shelf hardware components such as an integrated camera system with CMOS sensor with microphone (MC900, third eye electronics, dynamic range: 48 dB) and an Arduino Mega board for hardware control. It has a field-of-view of 800 by 600 μm (640 by 480 pixels) using an optical design similar to that of the first-generation UCLA Miniscope with a GRIN lens as an objective and an achromatic lens to focus an image on the CMOS imaging sensor. The camera sensor permits acquisitions at 30 Hz and the use of 3D printed components allows for rapid prototyping and lighter designs at a reduced cost relative to machined parts. This FinchScope incorporates a threaded turret for focusing, weighs approximately 1.8 g when used in a wired configuration and can be combined with a specially designed active commutator that allows for longitudinal observations of bird song in zebra finches. The software permits low-latency feedback to trigger recording during bird vocalization and was crucial in the discovery of neural dynamics underlying stable motor patterns (Liberti et al., 2016). With an added 2 g wireless transmitter and lithium-polymer battery, the FinchScope is capable of fully wireless recording and data transmission. The extra weight favors its application in larger animals capable of carrying the additional weight and the developers have claimed success using the wireless version in mice.

### miniScope

The miniScope project developed at the National Institute on Drug Abuse (NIDA) is a 2.4 g miniscope that has been used to study direct and indirect pathway of the striatum, investigating how activity correlates with ongoing locomotory behavior (Barbera et al., 2016) as well as how activity patterns in medial prefrontal cortex associate with social salience and novelty during behavior (Liang et al., 2018; Zhang et al., 2018). The miniScope housing was 3D printed externally using SLArmor with nickel plating, a strong, light and light-tight material (Protolabs). Like the FinchScope, the miniScope has a threaded turret for focusing. It uses an achromatic doublet lens for focusing onto a CMOS imaging sensor and an aspheric lens in combination with a relay GRIN lens to reach deep targets. The field-of-view is large at 1.1 by 1.1 mm and projected on a 400 by 400 pixel surface of a standard CMOS sensor (MT9V022IA7ATM, On Semiconductor, dynamic range > 55 dB) with a frame rate set to 10 Hz. Data acquisition occurs through an Opal Kelly field programmable gate array (FPGA) board which provides fast control over read-out of video data and control of the excitation LED light source.

### CHEndoscope

At the University of Toronto, a miniscope design named the CHendoscope has been developed. This miniscope has a housing that can be 3D printed (Jacob et al., 2018b) and is used with a 1.5 g 5 Megapixel integrated camera module (MU9PC-MBRD, Ximea, dynamic range: 38 dB) bringing the total weight of the scope to 4.5 g. To achieve 20 Hz sampling pixel binning is used to reach an effective imaging area of 648 by 486 pixels, corresponding to a field-of-view of approximately 500 μm across. Focusing is achieved through a threaded turret as in the FinchScope and miniScope. The power source to the miniscope has to be adjusted manually while video data is acquired through a Python software interface installed on a standard PC. The simplicity of the design, which uses commercially available imaging hardware like that of the FinchScope, makes it an attractive option for researchers that prefer to use off-the-shelf components rather than custom printed circuit boards (PCBs).

The miniscopes discussed above have variable ease of assembly, form factor and functionality, catering to specific needs of the researchers involved. Due to the open-source nature of these projects and their relatively simple components, this is not a major issue since modifications can be easily implemented making the development cycle short. Below we will focus on some of the ways in which miniscope functionality can be further improved.

## Improving Functionality of Open-Source Miniscopes

### Electrowetting Lenses

**Figure 3:**
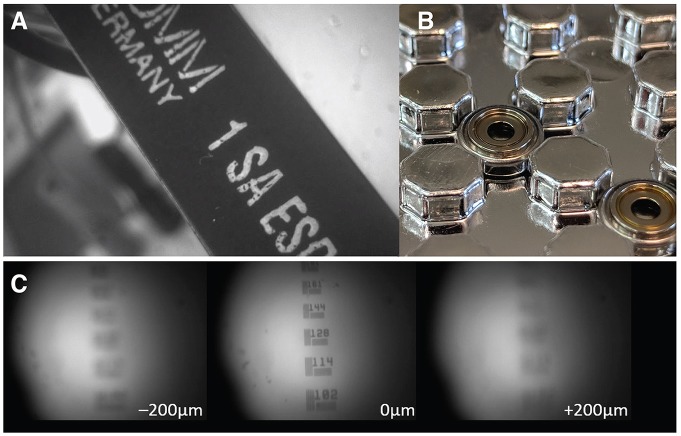
Electrowetting Lenses (EWLs). **(A)** Image of an object brought into focus with an EWL. **(B)** Commercially available miniature EWLs weigh as little as 0.2 g with an outer diameter of ~6 mm. **(C)** Example use of an EWL (Varioptic Arctic 25H0) to focus onto a 1951 USAF test target with a ±200 μm focal length shift.

Electrowetting lenses (EWLs, a.k.a. Liquid lenses) consist of an interface of two immiscible liquids (typically water and oil) which, under voltage, deform and act as a lens allowing for electronic focusing without the need for manual adjustment (Figure 3). This is especially important for longitudinal studies where an experimenter needs to recover the previously used focal plane and the same miniscope is either used across multiple animals or different regions in the same animal. Moreover, the reduced need for mechanical handling of sensitive components of the miniscope ensures a longer shelf life. An additional benefit is the optical power of EWLs can be adjusted rapidly, within the typical frame rate used with miniscopes (30 Hz), which can be leveraged if one wants to perform interleaved recording from different focal planes. Commercial vendors have begun to implement electronic focusing in their miniscopes and the miniaturization and availability of these lenses (e.g., Varioptic Arctic 16F0) has allowed their incorporation in open-source miniscopes. For example, a head-mounted two-photon excitation fiber-coupled microscope has incorporated an EWL to allow multi-plane recordings in unrestrained mice (Ozbay et al., 2018) and one-photon excitation based miniscopes will follow suit.

### Two-Color Fluorescence

**Figure 4:**
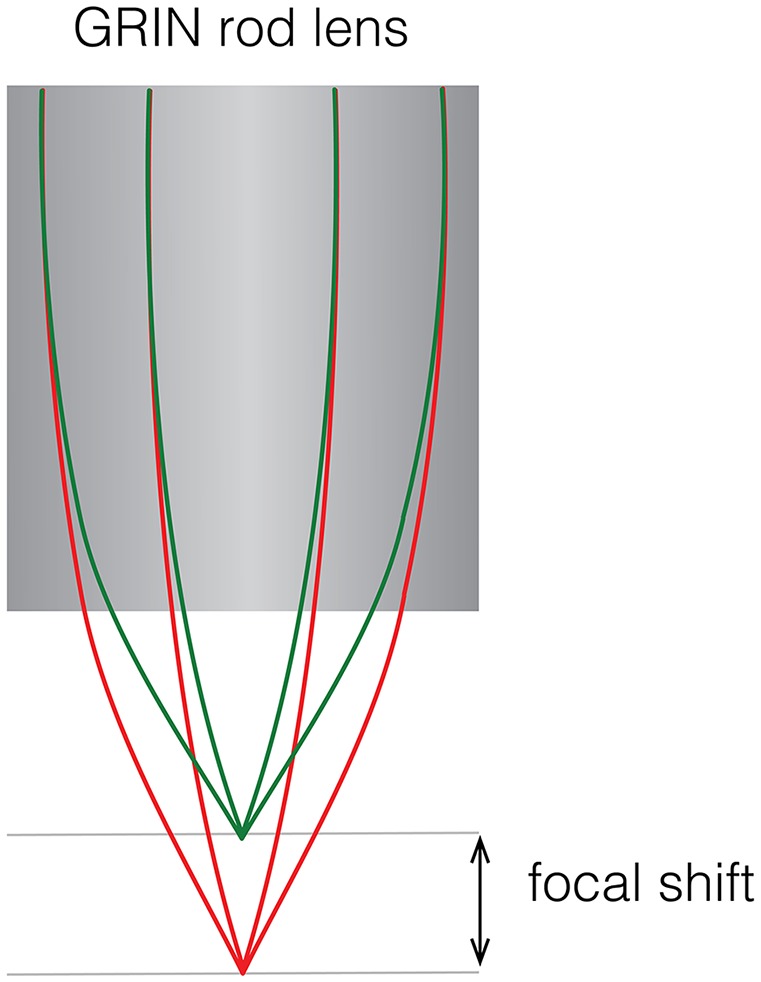
Focal shift caused by chromatic aberrations. Schematic showing focal shift in a GRIN rod lens when imaging two colors.

With the availability of genetically encoded activity reporters that fluoresce at spectrally separable wavelengths (Chen et al., 2013; Dana et al., 2016), it becomes possible to target distinct cell populations for imaging, providing insight into how the dynamics of two neuronal populations are correlated to specific behaviors (Jennings et al., 2019). One way to implement two-color imaging in a miniscope is to add an additional dichroic mirror in the emission path to split fluorescent light from two fluorophores onto two detectors which can independently positioned. This solution, which has been implemented by a commercial vendor of miniaturized microscopes, bypasses the problem of chromatic aberration that is inherent in GRIN lenses. Such aberrations introduce a focal shift (up to ~100 μm for larger diameter GRIN lenses and depending on the optical configuration) that cause fluorescence of distinct wavelengths to be sampled from separate focal planes (Figure 4). Alternatively, optical elements can be used to correct for aberrations, either by positioning a plano-convex lens directly behind a shortened GRIN lens (Leiner and Prescott, 1983), by entirely replacing the objective GRIN lens with an achromatic stack of lenses, or by using diffractive optical elements (requiring custom optics). Simultaneous two-color imaging can also be achieved using a single imaging sensor with a modified miniscope optical path. Two alternating light sources combined with two band pass filters and a dichroic are used to generate and combine spectrally separated excitation. A dual-band pass dichroic and emission filter are used in the emission path to transmit emitted fluorescence. Detection of the distinct fluorescent wavelengths is achieved through interleaved read-out from a single sensor synchronized with the alternating light sources. Color imaging sensors, which use an RGB Bayer filter, can also be used for imaging two fluorophores but with the tradeoff of image resolution for spectral information and often require additional offline processing to isolate the distinct wavelengths from the spectrally overlapping RGB recording. The first forays into the development of open-source dual color miniscopes have already begun and led to open-source two-color miniscopes with some correcting for chromatic aberrations (Jacob et al., 2018a; Leman et al., 2018). While chromatic aberration correction can be implemented by replacing the objective GRIN lens with achromatic optics, accessing deeper-lying structures may still require a relay GRIN lens which introduces its own aberrations.

### Optogenetics

By using light-driven channels that have minimally overlapping spectra with a fluorescent activity reporter, it is possible to build a miniscope with concurrent neural imaging and manipulation capabilities in the field-of-view (Stamatakis et al., 2018). This can be achieved using a dual excitation path similar to the one described in the previous section. Depending on the opsin and fluorescent activity reporter chosen, these optogenetic capable miniscopes can have reduced, but not fully removed, cross-talk of between channels. For example, Stamatakis et al. showed in their commercial implementation of a miniaturized optogenetic microscope that the imaging light source attenuates the optogenetic response that could be evoked in a slice test preparation. This demonstrated that one should proceed cautiously when adjusting the excitation light source for imaging to provide a good trade-off between signal-to-noise and minimal signal crossover. An alternative approach is to stimulate away from the imaging field-of-view which is possible by implanting optical fibers or wireless implantable μLEDs (Shin et al., 2017). However, this requires a distinct LED driver that is synchronized with the miniscope. An alternative implementation could use an additional LED driver on the miniscope itself in combination with an implant for optogenetic stimulation (De Groot et al., 2018).

### Wide-Field Imaging

**Figure 5:**
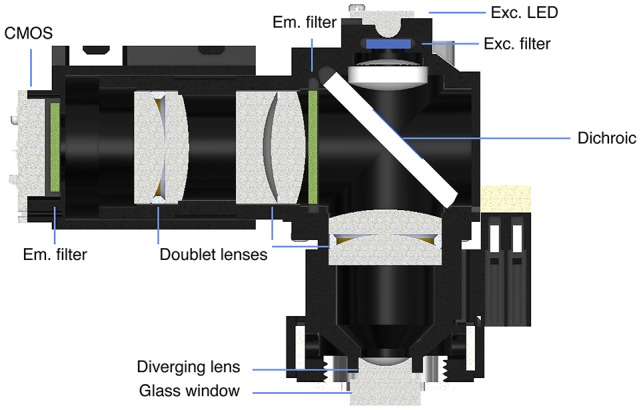
cScope macroscope developed at Princeton University. Schematic showing the optics used to increase the field-of-view for a head-mounted microscope. Schematic courtesy S.Y. Thiberge, Princeton University.

Until recently, the fields-of-view of existing open-source miniscopes with single cell resolution extended up to a maximum reported field-of-view of ~1 by 1 mm using an achromatic objective (Zhang et al., 2018). In the realm of relatively small footprint microscopes that can be carried by mice, larger imaging sensors could be combined with a set of plano-convex lenses and double concave lens to achieve a larger (3 by 4 mm^2^) field-of-view (Leman et al., 2018). For imaging applications in vertebrates larger than mice, the cScope (33 g) developed at Princeton University^1^ provides the option to significantly expand the field-of-view (to 7.8 mm × 4 mm) and investigate intra-cortical activity patterns during free-roaming behavior at less than single cell resolution (Scott et al., 2018). Making use of the UCLA Miniscope imaging and data acquisition electronics, the cScope’s large field-of-view was achieved through optimized macroscope optics and collating images taken at different angular positions of the imaging sensor (Figure 5). In general, by replacing the objective GRIN lens and a selection of aberration-corrected lens elements, a wider field of view can be obtained: a trend that we will see implemented in future open-source miniscopes.

### Neurovascular Coupling

Hemodynamic signals contaminate fluorescence signals imaged with an epifluorescence microscope. In addition to a 480 nm LED light source for fluorescence imaging, the cScope microscope design integrates LED illumination (530 nm) to measure the intrinsic optical signal (OIS). The latter provides a measure of blood oxygenation that can be used to correct the fluorescence signal originating from neural activity. The cScope can thus be used not only to monitor neuronal activity in large areas of the brain and subtract the hemodynamic signal, but also provide insights into neurovascular coupling. To study this in mice, a 9-g miniscope—with a weight-relief system limiting the head-borne weight to 3 g—has recently been developed (Senarathna et al., 2019). This microscope uses a 452 nm LED for fluorescence excitation, a 570 nm LED light for OIS imaging and a 680 nm laser diode for deoxyhemoglobin absorption measurements. A set of orange LEDs is used for signal synchronization of the scope with peripheral hardware.

### Volumetric Imaging

The principle underlying light-field microscopy is to collect angular information along with positional information of light rays emitting from a volume (e.g., their intensity, direction, wavelength, or polarization) and use computational methods to reconstruct volumetric information *post hoc* from information contained in a 2D image (Cohen et al., 2014; Prevedel et al., 2016). One common implementation of light-field microscopy uses a micro lens array (MLA) to focus light coming from different angles onto different pixels of the imaging sensor. An MLA was implemented in a reconfigured UCLA Miniscope (MiniFLM) to achieve a 700 by 600 by 360 μm^3^ volume that could be acquired at 16 Hz (Skocek et al., 2018). An iterative source extraction procedure (Seed-Iterative Demixing, SID) was developed specifically for use with light-field imaging in scattering tissue (Nöbauer et al., 2017) and could be used in combination with the MiniLFM microscope to reliably extract signals from neurons separated by 15 μm or more. Some complexities arise using MiniFLM given that it is harder to correct for motion artifacts and some spatial information is traded for angular information but the overall increase in the amount of data that can be collected when imaging volumes at video frame rates (e.g., in combination with cell-specific targeting of activity reporters) can be significant compared to wide-field imaging.

### Wireless and Wire-Free Recordings

Wireless (Liberti et al., 2017) and wire-free (Shuman et al., 2018) open-source miniscopes have been developed. Both are powered by onboard lithium polymer batteries which add considerable weight to the microscope design. Power consumption scales with data bandwidth and is one of the limiting factors in designing a miniaturized wireless microscope due to battery size and weight requirements. Power efficient CMOS imaging sensors can be used along with pixel binning or pixel subsampling to minimize power consumption while maintaining a comparable field of view to wired miniscopes. Most imaging sensors used in miniaturized microscopes have a higher pixel density than required when compared with the calculated point spread function for their optical path and a reduced spatial resolution from scattering in tissue. Thus, lower or downsampled sensor resolution is chosen to extend recording time while maintaining acceptable weight requirements. In addition, wireless powering of a miniaturized design with low power consumption is feasible with near-field wireless power transfer similar to those that have already been implemented for wireless optogenetic stimulation (Montgomery et al., 2015; Park et al., 2015; Shin et al., 2017).

### Electrophysiology

As part of the UCLA Miniscope project various miniscope implementations are under development that support simultaneous extracellular recordings and imaging. This can be achieved through a standalone ephys recording system synchronized to a miniscope or by fully incorporating ephys recording hardware into the Miniscope system using digital electrophysiology interface chips (e.g., Intan’s RHD2132/64). For rats, an assembly for microdrive recordings of up to 18 channels (OvalDrive 18-ES) has been developed at UCLA^2^, which allows for simultaneous electrophysiological recordings or voltammetry and imaging in rats. Moreover, fluid injection cannulae and optical fibers can also be inserted enabling manipulation of activity during miniscope imaging in freely-moving rats.

### Behavioral Read-Out

Miniscope recordings can be combined with cameras that record animal behavior, but storing, processing and annotating such behavioral datasets is often nontrivial and time-consuming. A framework to process data streams named Bonsai (Lopes et al., 2015) aids in automating parts of such analysis and a module has been developed to support the UCLA Miniscope^3^. An alternate way to log the behavioral state of the animal is to integrate an inertial measurement unit (IMU) in a miniscope to measure 3D head acceleration and orientation (De Groot et al., 2018). Grooming, eating and rearing behaviors are associated with distinct patterns of acceleration that can be leveraged to classify behavior (Venkatraman et al., 2010; Pasquet et al., 2016; Wilson et al., 2018). For more sophisticated behavioral tracking multi-camera systems have been used to e.g., track 3D posture (Mimica et al., 2018) but require significant resources that may not be accessible to all labs. Another tracking framework (3D Tracker)^4^ makes use of four RealSense^TM^ depth sensing cameras and is relatively low-cost and effective for tracking posture and social interactions in rats but requires significant post-processing (Matsumoto et al., 2013; Nakamura et al., 2016). Advances in machine learning will lead to an automated analysis of behavioral motifs captured in camera data such as in the extraction of the temporal structure of animal pose (Wiltschko et al., 2015; Pereira et al., 2019) or to markerless animal tracking (Mathis et al., 2018). Open Ephys, which develops open-source tools for neuroscience^5^, has stepped forward with a solution for behavioral tracking using tracking systems originally developed for consumer gaming: Valve’s SteamVR^TM^ tracking. It makes use of orthogonal laser emitters in a base station that sweep space (two sweeps 2pi/360°) along with photodiodes and an IMU on the tracked object (e.g., miniscope) to derive information about a tracked objects orientation, velocity and angular velocity in real-time. This system is advantageous in that it does not require the same amount of post-processing as in the aforementioned systems, is affordable and can be integrated with other Open Ephys peripherals.

### Two-Photon Excitation

Two-photon excitation offers well-known benefits over one-photon excitation, including a small excitation volume, increased tissue-penetration and reduced phototoxicity due to the use of longer excitation wavelengths. The small femtoliter excitation volume drastically improves the optical sectioning of the sample and thereby ensures that the light collected is only from the cellular and subcellular structures that lie along the focal plane. The first documented attempt to built a fiber-based miniaturized two-photon excitation microscope (Helmchen et al., 2001) demonstrated its overall feasibility, even though no functional imaging of neurons was achieved during behavior, in part due to strong motion artifacts. The microscope was also comparatively heavy and large (25 g, 70 mm high) limiting applications involving natural behavior in small animals. Initially, a piezoelectric element was used to drive vibrations in a stiffened fiber to generate a Lissajous scan pattern (Helmchen, 2002; Flusberg et al., 2005, 2008). Further improvements based on a similar design led to significantly lighter portable two-photon excitation microscopes (Göbel et al., 2004; Flusberg et al., 2008; Sawinski et al., 2009) used to image in freely moving mice and rats (see also Ozbay et al., 2018). Alternative scan designs based on micro-electro-mechanical systems (MEMS) mirrors have also been developed (Piyawattanametha et al., 2006; Zong et al., 2017). Amongst those, a portable two-photon excitation scanning microscope weighing near 2 g has been used to study the entorhinal cortex in freely behaving mice (Zong et al., 2017; Obenhaus et al., 2018). Expensive table-top lasers are used to achieve two-photon excitation and are coupled into one optical fiber while fluorescence is collected through an optical fiber for detection. The cost of a high-frequency pulsed laser and other peripherals (e.g., photomultiplier tubes, custom-made optical fibers for increased collection efficiency and flexibility) may limit the ubiquitous use of two-photon relative to one-photon excitation miniaturized microscopes. Furthermore, a recent comprehensive study pitching two-photon against one-photon excitation calcium imaging using respectively a benchtop two-photon and a miniaturized one photon excitation microscope revealed that orientation tuning of the same identified neurons was comparable irrespective of the type of fluorescence excitation used (Glas et al., 2018). Thus, despite the key advantages of two-photon excitation microscopy, miniaturized one-photon excitation fluorescence microscopes remain useful, affordable tools for cellular resolution imaging during unrestrained behavior. This holds even more so for highly mobile animals such as birds and bats, where fiber coupling of the excitation and emission light is impractical.

### Data Analysis

The brains of animals move during free-roaming behavior, which means that motion-correction is required prior to signal extraction. Although different types of motion correction algorithms have been implemented (Dombeck et al., 2007; Greenberg and Kerr, 2009; Dubbs et al., 2016; Mitani and Komiyama, 2018), a piecewise rigid implementation^6^ adapted for fluorescence endoscopic data has proven particularly effective in correcting non-rigid deformations (Pnevmatikakis and Giovannucci, 2017). A more recent approach applying a hierarchical video registration framework to distinguish stable frames from those with large inter-frame shifts could provide even more robust image registration (Lu et al., 2018). Once frames are registered, signals need to be extracted and demixed from detected cells. Initially a PCA/ICA approach was used for data obtained with miniaturized fluorescence microscopes (Mukamel et al., 2009) but this approach has been mostly replaced by more accurate methods based on CNMF with an added model to estimate and account for time-varying background fluctuations in fluorescence entitled CNMF-E (Zhou et al., 2018). It has been proposed that, although accurate in finding cells, the CNMF-E algorithm^7^ may have a higher false positive rate than the MIN1PIPE approach^8^ developed by Lu et al. (2018) which uses an alternate method to remove background fluctuations in fluorescence and enhance neural signals. The signal output is typically a scaled version of the fractional change in fluorescence, which can be normalized by dividing by the standard deviation or a baseline noise estimate. Deconvolution on the raw signal is often performed to denoise the signal and allow estimation of event times, which reflect underlying neural spiking activity.

## Future Developments

The open-source miniscope community is at the verge of releasing a wide variety of second-generation miniscopes that incorporate ETLs for focusing, two-color fluorescence detection, larger field-of-views, smaller footprints and optogenetic and behavioral read-out capabilities. What sort of developments lie ahead?

### Closed-Loop Experiments

The availability of FPGA boards (Ciliberti and Kloosterman, 2017; Siegle et al., 2017; Buccino et al., 2018) enables fast closed-loop experiments (Ahrens et al., 2012; Clancy et al., 2014; Packer et al., 2014; Prsa et al., 2017) providing insights into e.g., sensorimotor and prosthetic learning in neural circuits, which are relevant for the development of brain-machine interfaces. Real-time detection of a specific type of behavior or activity pattern can be used to directly manipulate input pathways or targeted cell assemblies. The cellular substrates underlying the expression of behavior or the interactions between brain, body and environment could then be investigated (Buckley and Toyoizumi, 2018). Closed-loop systems could also be utilized to detect and suppress the development of epileptiform activity (Sorokin et al., 2017) and delineate the cellular mechanisms underlying effective deep-brain stimulation (Parastarfeizabadi and Kouzani, 2017; Ghasemi et al., 2018). Open-source frameworks like Open Ephys have already developed hardware integrating FPGAs for low latency closed-loop experiments (Siegle et al., 2017) and such closed-loop capabilities could be ported to open-source miniscopes. Given the expansion of miniscope functionality and the concomitant increase in data channels that need to be processed, the use of FPGAs would streamline data processing and control. With miniscopes permitting volume imaging and the need for signal demultiplexing such technology could also aid efficient data extraction.

### Custom Optics

The use of a set of achromatic lenses rather than an objective GRIN lens helps to reduce, but not fully remove, chromatic aberrations in most two-color miniscope configurations. One solution is the use of a diffractive element or axicon that corrects the chromatic aberration in the optical path. Nanolithography allows for the 3D printing of custom optical elements including ones that correct chromatic aberrations (Schmid et al., 2018), collimate light (Thiele et al., 2016) and act as high-resolution imaging lenses (Gissibl et al., 2016). The miniaturization of these optical components, when combined with a small footprint CMOS sensor, could lead to significantly lighter and smaller miniscopes enabling recording from multiple regions concurrently using more than one head-mounted miniscope. Moreover, resonant plasmonic metasurfaces in combination with phase-change materials can be used to create miniaturized nano-optical elements than act as bifocal zoom lenses and can steer light (Yin et al., 2017). Three dimensional printing of optics still relies on expensive two-photon lasers that build-up components layer by layer in a time-consuming process, limiting their present widespread use in open-source miniscopes. However, a number of academic institutions have begun to provide a fee-for-service nanolithography infrastructure that could make this technology sufficiently mainstream to commence integration of 3D printed optical elements in miniscopes. Moreover, once a final 3D printed optical design has been tested, custom-machined optics inspired by these designs can be ordered through specialized contractors for micro-optics.

### Lens-Free Imaging

Important strides towards further miniaturization of imaging devices for fluorescence microscopy are being made by replacing focusing elements in miniscopes with a diffuser or mask in the fluorescence emission path (Adams et al., 2017; Antipa et al., 2017). A diffusive element with an optimized amplitude mask in combination with computational signal unmixing permits reconstruction of signals from a volume based on information contained in 2D images (similar to what has been used for light-field imaging incorporating MLAs). Lensless miniscopes have the potential to significantly reduce the weight and footprint of head-mounted imaging systems while also increasing their field-of-view. One of the caveats at present is that light sources need to be integrated in the system to allow for fluorescence excitation. This could be done in principle by positioning μLEDs around the imaging sensor or by implanting optical waveguides which can produce steerable light beams and sheets. Another caveat is that, as in the case of the MiniLFM microscope, motion correction can be hard to implement. However, the idea of a lensless miniscope holds a lot of promise and could lead to much lighter, large-field portable fluorescence microscopes.

### High Frame-Rate Acquisition

Most miniscopes at present are used in combination with GECIs, which have—in addition to Ca^2+^ providing an indirect measure of ongoing activity—relatively slow kinetics. Thus, here typical frame rates of 30 Hz are generally sufficient. With the rapid improvement of genetically encoded voltage indicators and their potential for cell-specific targeting *in vivo* (Flytzanis et al., 2014; Marshall et al., 2016; Piatkevich et al., 2018; Bando et al., 2019; Quicke et al., 2019) it becomes relevant to achieve higher frame rates with high sensitivity imaging sensors. Currently available CMOS image sensors sufficiently small to integrate in miniscopes can oftentimes reach frame rates of several 100 Hz, sufficient to look at population level voltage oscillations (Marshall et al., 2016), through a combination of pixel subsampling, pixel binning, and reducing the number of active pixel rows. To achieve single cell and single action potential resolution, likely both brighter voltage indicators and smaller back illuminated scientific CMOS sensors will need to be developed. Given further electronics miniaturization, heating is an issue that needs to be resolved. Heat can be dissipated by providing sufficient air convection or through metal heat sinks, both of which could add significant weight to a miniscope design. Operating at higher sampling frequencies will consume more power and generally require additional heat dissipation, setting a limit on using these high frame rates particularly in wire-free or wireless configurations.

## Concluding Remarks

The development of open-source miniscopes has given a big boost to neuroscientists that want accessible, affordable and understandable tools to image the vertebrate brain during naturalistic behavior. Open-source designs typically have relatively simple design plans with modular components that are readily available or can be custom-fabricated using 3D printers. The low cost of these components and the comparative ease with which such scopes can be assembled have led to their widespread adaptation. While many of these systems are straightforward enough to be disseminated and implemented rather easily, it remains a challenge to integrate more complex, less mainstream technologies into open-source miniscopes at scale. Luckily, access to affordable, high quality, low quantity production lines for custom PCBs, optics, CNC machining and injection molded parts has seen significant growth in recent years and is likely to continue. Components that were bulky and expensive only a few years ago have now been miniaturized and are affordable such as EWLs for rapid electrical focusing, higher speed back-illuminated CMOS sensors and low-power IMUs that can be integrated on miniscopes to read-out head acceleration and orientation. Following this trend, even technologies such as nanolithography that seem somewhat exotic at present for an open-source development project may be more a commonplace in a few years. The ability to build lightweight small footprint miniscopes will contribute to animal well-being and improve the read-out of neural activity under natural conditions. The open-source community has strength in numbers and the continued open exchange of new ideas will lead to improved miniscopes (e.g., sensitivity, field-of-view, and closed-loop experimental control), broader dissemination and a better understanding of the brain as it interacts with the environment.

## Author Contributions

DA and TH prepared the figures and wrote the manuscript.

## Conflict of Interest Statement

The authors declare that the research was conducted in the absence of any commercial or financial relationships that could be construed as a potential conflict of interest.

## Footnotes

^1^https://github.com/PrincetonUniversity/cScope

^2^https://goo.gl/aJxJQy

^3^https://github.com/jonnew/Bonsai.Miniscope

^4^http://www.3dtracker.org

^5^http://www.open-ephys.org/

^6^https://github.com/flatironinstitute/NoRMCorre

^7^https://github.com/zhoupc/CNMF_E

^8^https://github.com/JinghaoLu/MIN1PIPE
